# Supplementary material for: Niche construction mediates climate effects on recovery of tundra heathlands after extreme event
Source: PLoS One. 2021 Feb 4;16(2):e0245929. doi: 10.1371/journal.pone.0245929 (PMC7861441; doi:10.1371/journal.pone.0245929)
Supplement: S1 Fig — Continentality index per site is presented in parenthesis. Species included Avenella flexuosa, Solidago virgaurea, Calamagrostis phragmitoides, Chamerion angustifolium, Bistorta vivipara, Rhinantus minor and Melampyrun pratense. (DOCX) [file pone.0245929.s001.docx]

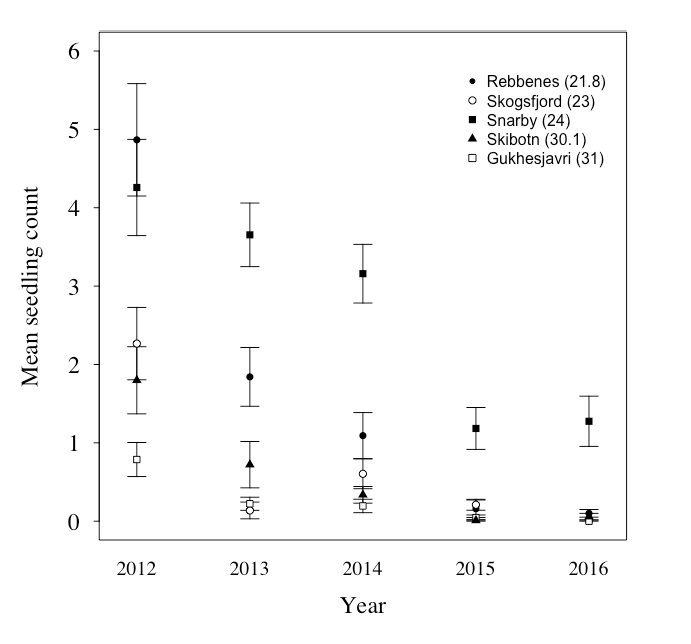
S1 Fig. **Mean seedling count from seed sowing experiment per site (± 95 % confidence intervals) between 2012-2016 across all sites**. Continentality index per site is presented in parenthesis. Species included *Avenella flexuosa*, *Solidago virgaurea ,* *Calamagrostis phragmitoides,* *Chamerion angustifolium* , *Bistorta vivipara* , *Rhinantus minor* and *Melampyrun pratense.*
