# Supplementary material for: Niche construction mediates climate effects on recovery of tundra heathlands after extreme event
Source: PLoS One. 2021 Feb 4;16(2):e0245929. doi: 10.1371/journal.pone.0245929 (PMC7861441; doi:10.1371/journal.pone.0245929)
Supplement: S2 Fig — Species used were Avenella flexuosa, Rumex acetosa and Solidago virgaurea growing for three weeks in each of the three soil types (i.e. sampled from control plots (C), from extreme event plots (EE) in 2016, and commercially available peat (Peat)). (DOCX) [file pone.0245929.s002.docx]

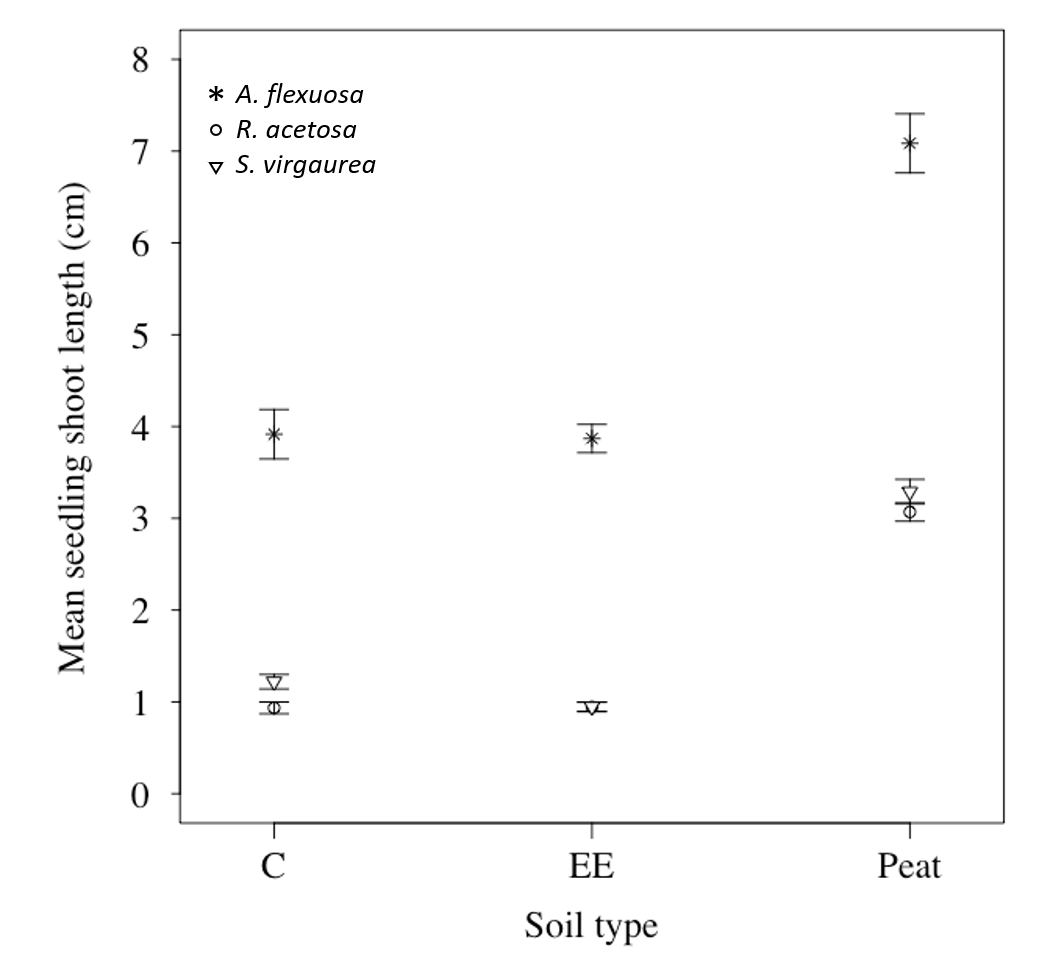


S2 Fig. **Mean shoot length from bioassay experiment (± 95 % confidence intervals)**. Species used were *Avenella flexuosa, Rumex acetosa* and *Solidago virgaurea* growing for three weeks in each of the three soil types (i.e. sampled from control plots (C), from extreme event plots (EE) in 2016, and commercially available peat (Peat)).
