# Supplementary material for: Niche construction mediates climate effects on recovery of tundra heathlands after extreme event
Source: PLoS One. 2021 Feb 4;16(2):e0245929. doi: 10.1371/journal.pone.0245929 (PMC7861441; doi:10.1371/journal.pone.0245929)
Supplement: S1 Table — (DOCX) [file pone.0245929.s003.docx]

S1 Table. List of vascular plant species and growth forms registered in extreme event and control plots in 2019 in order of decreasing biomass (g/m^2^).

| Extreme event plots | Growth form | Control plots | Growth form |
| --- | --- | --- | --- |
| *E. nigrum nigrum* | Evergreen dwarf shrub | *E. nigrum nigrum* | Evergreen dwarf shrub |
| *Betula nana* | Decidious dwarf shrub | *Betula nana* | Decidious dwarf shrub |
| *Vaccinium vitis-idaea* | Evergreen dwarf shrub | *Vaccinium uluginosum* | Decidious dwarf shrub |
| *Vaccinium myrtillus* | Decidious dwarf shrub | *Vaccinium myrtillus* | Decidious dwarf shrub |
| *Vaccinium uluginosum* | Decidious dwarf shrub | *Vaccinium vitis-idaea* | Evergreen dwarf shrub |
| *Chamaepericlymenum suecica* | Decidious dwarf shrub | *Chamaepericlymenum suecica* | Decidious dwarf shrub |
| *Andromeda polifolia* | Evergreen dwarf shrub | *Avenella flexuosa* | Grass |
| *Loiselaurea procumbens* | Evergreen dwarf shrub | *Loiselaurea procumbens* | Evergreen dwarf shrub |
| *Avenella flexuosa* | Grass | *Andromeda polifolia* | Evergreen dwarf shrub |
| *Nardus stricta* | Grass | *Arctostaphyllus alpinum* | Decidious dwarf shrub |
| *Carex_bigelowii* | Sedge | *Nardus_stricta* | Grass |
| *Arctostaphyllus alpinum* | Decidious dwarf shrub | *Carex_bigelowii* | Sedge |
| *Salix herbacea* | Decidious dwarf shrub | *Calluna vulgaris* | Evergreen dwarf shrub |
| *Eriophorum* sp. | Sedge | *Diapensia lapponica* | Evergreen dwarf shrub |
| *Rubus chamaemorus* | Herbaceous | *Rubus chamaemorus* | Herbaceous |
| *Calamagrostis neglecta* | Grass | *Eriophorum* sp. | Sedge |
| *Pedicularis palustris* | Herbaceous | *Salix herbacea* | Decidious dwarf shrub |
| *Trientalis_europaea* | Herbaceous | *Juncus* sp. | Rush |
| *Diapensia lapponica* | Evergreen dwarf shrub | *Calamagrostis neglecta* | Grass |
| *Calluna vulgaris* | Evergreen dwarf shrub | *Trientalis europaea* | Herbaceous |
| *Juncus* sp. | Rush | *Pedicularis palustris* | Herbaceous |
