# Supplementary material for: Niche construction mediates climate effects on recovery of tundra heathlands after extreme event
Source: PLoS One. 2021 Feb 4;16(2):e0245929. doi: 10.1371/journal.pone.0245929 (PMC7861441; doi:10.1371/journal.pone.0245929)
Supplement: S2 Table — Significant values are presented in bold. (DOCX) [file pone.0245929.s004.docx]

S2 Table. Anova tables from the linear models showing the effect of the extreme event (EE), Continentality index (CI) and their interactions (CIxEE) on the mean values of a) species richness, total biomass, biomass of subordinate species and biomass of *E. nigrum* alone; b) leaf traits: specific leaf area (SLA), leaf dry matter content (LDMC) and leaf nitrogen content (LNC), c) seedling length of the vascular plant species used in bioassay, and d) lichen, moss and soil. Significant values are presented in **bold.**

| a) | Species richness | | | Total biomass | | | Subordinate species | | | Mean frequency *E. nigrum* | | | |
| --- | --- | --- | --- | --- | --- | --- | --- | --- | --- | --- | --- | --- | --- |
|  | DF | F-value | p-value | DF | F-value | p-value | DF | F-value | p-value | DF | F-value | p-value |  |
| EE | 1,94 | 18.8 | **<0.01** | 1,94 | 691.6 | **<0.01** | 1,94 | 2.63 | 0.17 | 1,94 | 763.2 | **<0.01** |  |
| CI | 1,3 | 0.16 | 0.70 | 1,3 | 2.99 | 0.15 | 1,3 | 10.16 | **0.04** | 1,3 | 1.53 | 0.30 |  |
| CIxEE | 1,94 | 2.18 | 0.14 | 1,94 | 12.7 | **0.03** | 2,94 | 6.51 | **0.02** | 2,94 | 6.12 | **0.02** |  |

| b) | SLA | | | LDMC | | | LNC |  |  |
| --- | --- | --- | --- | --- | --- | --- | --- | --- | --- |
|  | DF | F-value | p-value | DF | F-value | p-value | DF | F-value | p-value |
| EE | 1,94 | 32.05 | **<0.01** | 1,94 | 9.01 | **<0.01** | 1,94 | 32.17 | **<0.01** |
| CI | 1,3 | 0.87 | 0.41 | 1,3 | 0.68 | 0.46 | 1,3 | 5.9 | 0.09 |
| EE x CI | 1,94 | 0.90 | 0.34 | 1,94 | 0.32 | 0.57 | 1,94 | 3.49 | 0.06 |

| c) | *A. flexuosa* | | | *S. virgaurea* | | | *R. acetosa* | | |
| --- | --- | --- | --- | --- | --- | --- | --- | --- | --- |
|  | DF | F-value | p-value | DF | F-value | p-value | DF | F-value | p-value |
| EE | 1,94 | 0.02 | 0.95 | 1,94 | 7.45 | **0.01** | 1,94 | 0.02 | 0.98 |
| CI | 1,3 | 0.24 | 0.65 | 1,3 | 0.0 | 0.99 | 1,3 | 0.03 | 0.87 |
| CIxEE | 1,94 | 1.63 | 0.21 | 2,94 | 0.0 | 0.96 | 2,94 | 3.33 | 0.08 |

| d) | Lichen | | | Moss | | | Soil | | |
| --- | --- | --- | --- | --- | --- | --- | --- | --- | --- |
|  | DF | F-value | p-value | DF | F-value | p-value | DF | F-value | p-value |
| EE | 1,94 | 11.16 | **<0.01** | 1,94 | 0.01 | 0.91 | 1,94 | 333.2 | **<0.01** |
| CI | 1,3 | 0.04 | 0.83 | 1,3 | 5.54 | 0.10 | 1,3 | 0.82 | 0.42 |
| CIxEE | 1,94 | 0.67 | 0.41 | 1,94 | 2.24 | 0.13 | 1,94 | 3.07 | 0.09 |
