# Supplementary material for: Niche construction mediates climate effects on recovery of tundra heathlands after extreme event
Source: PLoS One. 2021 Feb 4;16(2):e0245929. doi: 10.1371/journal.pone.0245929 (PMC7861441; doi:10.1371/journal.pone.0245929)
Supplement: S3 Table — Only significant interactions are presented. Significant values are presented in bold. (DOCX) [file pone.0245929.s005.docx]

S3 Table. ANOVA table from the linear model showing the effect of the extreme event (EE), Continentality index (CI), year, and the interaction of Year and Continentality index (YearxCI) on the mean seedling count of all species sown in 2011. Only significant interactions are presented. Significant values are presented in **bold**

|  | Mean Seedling Count | | |
| --- | --- | --- | --- |
|  | DF | F-value | p-value |
| EE | 1,142 | 5.59 | 0.20 |
| Year | 1,573 | 218.53 | **<0.01** |
| CI | 1,142 | 23.05 | **<0.01** |
| Year x CI | 1,573 | 36.54 | **<0.01** |
